# 2-Hy­droxy-*N*-(2-hy­droxy­eth­yl)benzamide

**DOI:** 10.1107/S1600536811029175

**Published:** 2011-07-23

**Authors:** Richard Betz, Thomas Gerber, Eric Hosten, Henk Schalekamp

**Affiliations:** a Nelson Mandela Metropolitan University, Summerstrand Campus, Department of Chemistry, University Way, Summerstrand, PO Box 77000, Port Elizabeth 6031, South Africa

## Abstract

In the title compound, C_9_H_11_NO_3_, a derivative of salicyl­amide, the intra­cyclic C—C—C angles span the range 117.96 (13)–121.56 (14)°. An intra­molecular O—H⋯O hydro­gen bond occurs. In the crystal, inter­molecular O—H⋯O and N—H⋯O hydrogen bonds occur and C—H⋯O contacts connect the mol­ecules into a three-dimensional network. The closest inter­centroid distance between two π-systems is 3.8809 (10) Å.

## Related literature

For the crystal structure of *N*-acetyl­salicyl­amide, see: Vyas *et al.* (1987 ▶). For graph-set analysis of hydrogen bonds, see: Etter *et al.* (1990 ▶); Bernstein *et al.* (1995 ▶). Structures containing similar dihedral angles were retrieved from the Cambridge Structural Database (Allen, 2002 ▶). For the use of chelating ligands in coordination chemistry, see: Gade (1998 ▶).
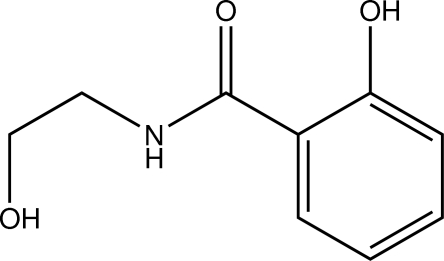


## Experimental

### 

#### Crystal data

C_9_H_11_NO_3_
*M* *_r_* = 181.19
Monoclinic, 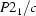

*a* = 8.5852 (5) Å
*b* = 12.1716 (7) Å
*c* = 9.1113 (4) Å
β = 115.682 (2)°
*V* = 858.04 (8) Å^3^
*Z* = 4
Mo *K*α radiation
μ = 0.11 mm^−1^
*T* = 200 K
0.39 × 0.14 × 0.13 mm

#### Data collection

Bruker APEXII CCD diffractometer
7333 measured reflections
2063 independent reflections
1561 reflections with *I* > 2σ(*I*)
*R*_int_ = 0.061

#### Refinement

*R*[*F* ^2^ > 2σ(*F* ^2^)] = 0.047
*wR*(*F* ^2^) = 0.113
*S* = 1.04
2063 reflections
130 parameters
H atoms treated by a mixture of independent and constrained refinement
Δρ_max_ = 0.26 e Å^−3^
Δρ_min_ = −0.21 e Å^−3^

### 

Data collection: *APEX2* (Bruker, 2010 ▶); cell refinement: *SAINT* (Bruker, 2010 ▶); data reduction: *SAINT*; program(s) used to solve structure: *SHELXS97* (Sheldrick, 2008 ▶); program(s) used to refine structure: *SHELXL97* (Sheldrick, 2008 ▶); molecular graphics: *ORTEP-3* (Farrugia, 1997 ▶) and *Mercury* (Macrae *et al.*, 2008 ▶); software used to prepare material for publication: *SHELXL97* and *PLATON* (Spek, 2009 ▶).

## Supplementary Material

Crystal structure: contains datablock(s) I, global. DOI: 10.1107/S1600536811029175/qm2017sup1.cif

Supplementary material file. DOI: 10.1107/S1600536811029175/qm2017Isup2.cdx

Structure factors: contains datablock(s) I. DOI: 10.1107/S1600536811029175/qm2017Isup3.hkl

Supplementary material file. DOI: 10.1107/S1600536811029175/qm2017Isup4.cml

Additional supplementary materials:  crystallographic information; 3D view; checkCIF report

## Figures and Tables

**Table 1:** Hydrogen-bond geometry (Å, °)

*D*—H⋯*A*	*D*—H	H⋯*A*	*D*⋯*A*	*D*—H⋯*A*
O2—H82⋯O1	0.90 (2)	1.73 (2)	2.5570 (15)	150 (2)
O3—H83⋯O1^i^	0.90 (2)	1.93 (2)	2.8197 (15)	168.3 (19)
N1—H71⋯O3^ii^	0.874 (18)	2.113 (19)	2.9697 (16)	166.6 (17)
C4—H4⋯O2^iii^	0.95	2.54	3.4864 (19)	173
C7—H7⋯O3^ii^	0.95	2.57	3.4496 (19)	155

Symmetry codes: (i) 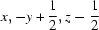; (ii) 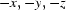; (iii) 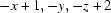.

**Fig. 1.**
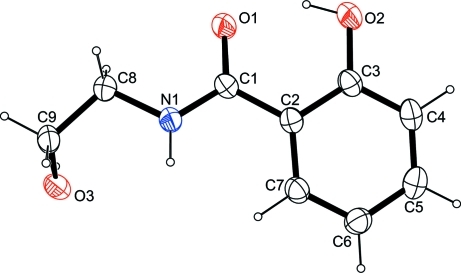
The molecular structure of the title compound, with atom labels and anisotropic displacement ellipsoids (drawn at 50% probability level).

**Fig. 2.**
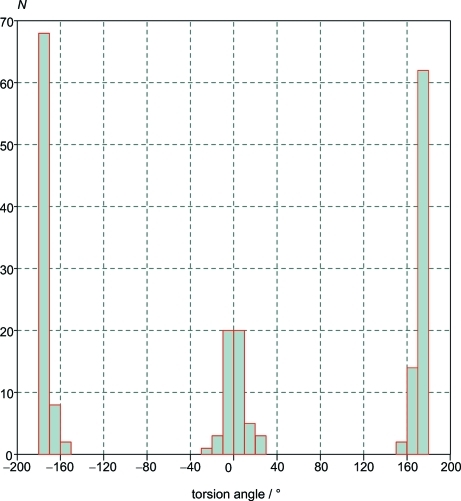
Statistical distribution of Car–Car–C=O dihedral angles in salicylic acid-derived amides (data based on CSD search including all structures up to November 2010).

**Fig. 3.**
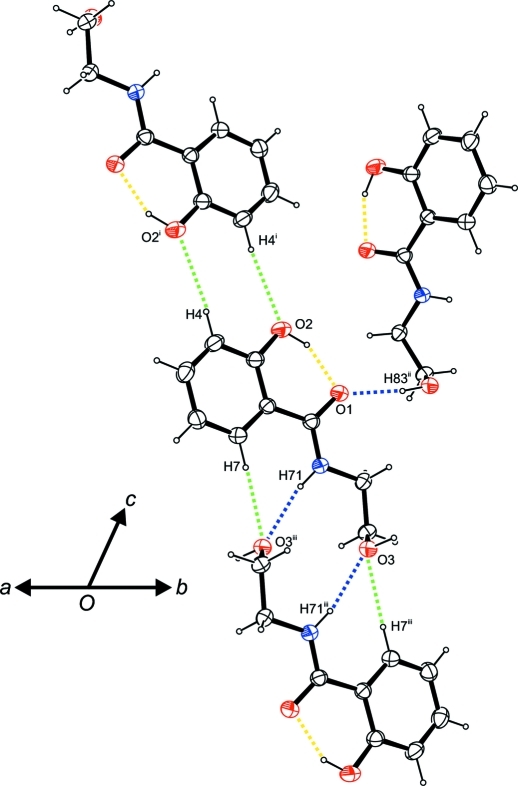
Intermolecular contacts, viewed along [-1 - 1 0] (green dashed lines: C–H···O contacts, yellow dashed lines: intramolecular hydrogen bonds, blue dashed lines: intermolecular hydrogen bonds). Symmetry operators: i -x + 1, -y, -z + 2; iix, -y + 1/2, z + 1/2; iii -x, -y, -z.

**Fig. 4.**
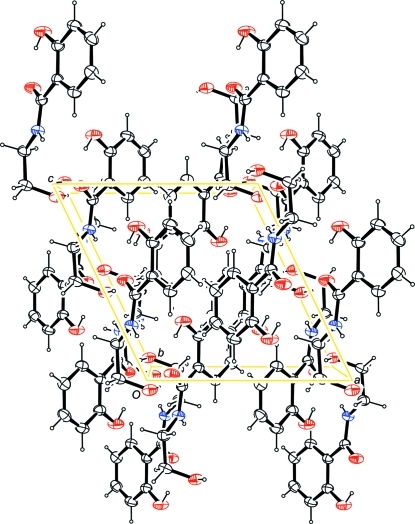
Molecular packing of the title compound, viewed along [0 1 0] (anisotropic displacement ellipsoids drawn at 50% probability level).

## supplementary crystallographic information

### Comment

Chelate ligands have found widespread use in coordination chemistry due to the enhanced thermodynamic stability of resultant coordination compounds in relation to coordination compounds exclusively applying comparable monodentate ligands (Gade, 1998). Combining different donor atoms, a molecular set-up to accomodate a large variety of metal centers of variable Lewis acidity is at hand. In this aspect, *N*-(2-hydroxyethyl)-salicylamide seemed of interest due to its possible use as a strictly neutral or, depending on the pH value, as an anionic or cationic ligand. In addition, due to the set-up of its functional groups, it may act as mono-, bi-, tri- or even tetradentate ligand offering the possibility to create chelate rings of various size. The intriguing combination of a secondary amino group, a keto group as well as an aliphatic and an aromatic hydroxyl group classifies the title compound as a highly versatile ligand. To enable comparative studies in terms of bond lengths and angles in envisioned coordination compounds, we determined the molecular and crystal structure of the title compound. Information about the crystal structure of *N*-acetylsalicylamide (Vyas *et al.*, 1987) is available in the literature.

Due to the possible resonance between the amide group and the aromatic system, a projection of the molecule shows nearly all atoms to reside in the same plane. The only marked exception from this finding is the aliphatic hydroxyl group which adopts a staggered conformation with respect to the plane of the phenyl moiety. Intracyclic C–C–C angles span a range from 117.96 (13)–121.56 (14) °. The least-squares planes defined by the carbon atoms of the aromatic system on the one hand and the CON-motif of the amide group on the other hand intersect at an angle of 11.71 (20) ° (Fig. 1). This finding is in good agreement with values reported for other salicylic acid-derived amides whose crystal structural data have been deposited with the Cambridge Structural Database (Allen, 2002; Fig. 2).

In the crystal structure, intra- as well as intermolecular hydrogen bonds are obvious. The intramolecular hydrogen bond is formed between the hydrogen atom of the hydroxyl group bonded to the aromatic system and the oxygen atom of the keto group, with the latter one also serving as acceptor for one intermolecular hydrogen bond stemming from the aliphatic hydroxyl group. The amino group acts as donor in a hydrogen bond applying the aliphatic hydroxyl group's oxygen atom as acceptor. Apart from these classical hydrogen bonds, C–H···O contacts are observed whose range falls by more than 0.1 below the sum of van-der-Waals radii of the atoms participating. These contacts are manifest between the CH group in *ortho* position to the hydroxyl group on the aromatic system and the O atom of this hydroxyl group in the neighbouring molecule thus connecting the molecules to centrosymmetric dimers. A second C–H···O contact can be observed between one of the aromatic CH groups and the O atom of the aliphatic hydroxyl group (Fig. 2). In terms of graph-set analysis (Etter *et al.*, 1990; Bernstein *et al.*, 1995), the descriptor for the classical hydrogen bonds is *S*(6)*C*^1^_1_(7)*R*^2^_2_(10) on the unitary level while a description of the C–H···O contatcs necessitates a *C*^1^_1_(8)*R*^2^_2_(8) descriptor on the same level. In total, the molecules are connected to a three-dimensional network. The closest intercentroid distance between two π-systems was found at 3.8809 (10) Å.

The packing of the title compound is shown in Figure 4.

### Experimental

The compound was obtained commercially (Aldrich). Crystals suitable for the X-ray diffraction study were taken directly from the provided compound.

### Refinement

Carbon-bound H atoms were placed in calculated positions (C—H 0.95 Å for aromatic C atoms and C—H 0.99 Å for methylene groups) and were included in the refinement in the riding model approximation, with *U*(H) set to 1.2*U*_eq_(C). The H atoms of the hydroxyl groups as well as the amine group were located on a difference Fourier map and refined with individual thermal parameters.

### Crystal data

**Table:**

C_9_H_11_NO_3_	*F*(000) = 384
*M**_r_* = 181.19	*D*_x_ = 1.403 Mg m^−^^3^
Monoclinic, *P*2_1_/*c*	Mo *K*α radiation, λ = 0.71073 Å
Hall symbol: -P 2ybc	Cell parameters from 3230 reflections
*a* = 8.5852 (5) Å	θ = 2.6–28.2°
*b* = 12.1716 (7) Å	µ = 0.11 mm^−^^1^
*c* = 9.1113 (4) Å	*T* = 200 K
β = 115.682 (2)°	Rod, colourless
*V* = 858.04 (8) Å^3^	0.39 × 0.14 × 0.13 mm
*Z* = 4	

### Data collection

**Table:**

Bruker APEXII CCD diffractometer	1561 reflections with *I* > 2σ(*I*)
Radiation source: fine-focus sealed tube	*R*_int_ = 0.061
graphite	θ_max_ = 28.0°, θ_min_ = 2.6°
φ and ω scans	*h* = −7→11
7333 measured reflections	*k* = −15→16
2063 independent reflections	*l* = −11→11

### Refinement

**Table:**

Refinement on *F*^2^	Primary atom site location: structure-invariant direct methods
Least-squares matrix: full	Secondary atom site location: difference Fourier map
*R*[*F*^2^ > 2σ(*F*^2^)] = 0.047	Hydrogen site location: inferred from neighbouring sites
*wR*(*F*^2^) = 0.113	H atoms treated by a mixture of independent and constrained refinement
*S* = 1.04	*w* = 1/[σ^2^(*F*_o_^2^) + (0.0456*P*)^2^ + 0.2715*P*] where *P* = (*F*_o_^2^ + 2*F*_c_^2^)/3
2063 reflections	(Δ/σ)_max_ < 0.001
130 parameters	Δρ_max_ = 0.26 e Å^−^^3^
0 restraints	Δρ_min_ = −0.21 e Å^−^^3^

### Fractional atomic coordinates and isotropic or equivalent isotropic displacement parameters (Å^2^)

**Table:**

	*x*	*y*	*z*	*U*_iso_*/*U*_eq_	
O1	0.12173 (15)	0.16491 (9)	0.50668 (12)	0.0345 (3)	
O2	0.30777 (16)	0.07953 (10)	0.78461 (13)	0.0385 (3)	
H82	0.234 (3)	0.1262 (18)	0.709 (3)	0.059 (6)*	
O3	−0.01603 (15)	0.12074 (9)	−0.05944 (13)	0.0320 (3)	
H83	0.031 (3)	0.188 (2)	−0.050 (3)	0.064 (7)*	
N1	0.05024 (16)	0.06865 (10)	0.27683 (15)	0.0265 (3)	
H71	0.055 (2)	0.0087 (15)	0.226 (2)	0.040 (5)*	
C1	0.14136 (19)	0.08091 (11)	0.43613 (17)	0.0245 (3)	
C2	0.26991 (18)	−0.00403 (11)	0.53026 (17)	0.0240 (3)	
C3	0.35056 (19)	0.00197 (12)	0.70163 (17)	0.0272 (3)	
C4	0.4777 (2)	−0.07369 (13)	0.79138 (19)	0.0325 (4)	
H4	0.5303	−0.0700	0.9069	0.039*	
C5	0.5277 (2)	−0.15370 (13)	0.7143 (2)	0.0341 (4)	
H5	0.6155	−0.2045	0.7768	0.041*	
C6	0.4508 (2)	−0.16085 (12)	0.54536 (19)	0.0317 (4)	
H6	0.4856	−0.2162	0.4923	0.038*	
C7	0.3235 (2)	−0.08696 (12)	0.45544 (18)	0.0283 (3)	
H7	0.2708	−0.0923	0.3400	0.034*	
C8	−0.0703 (2)	0.15326 (12)	0.17864 (18)	0.0280 (3)	
H8A	−0.0098	0.2248	0.1976	0.034*	
H8B	−0.1655	0.1603	0.2118	0.034*	
C9	−0.1441 (2)	0.12462 (13)	−0.00029 (18)	0.0310 (3)	
H9A	−0.2021	0.0523	−0.0179	0.037*	
H9B	−0.2324	0.1799	−0.0633	0.037*	

### Atomic displacement parameters (Å^2^)

**Table:**

	*U*^11^	*U*^22^	*U*^33^	*U*^12^	*U*^13^	*U*^23^
O1	0.0439 (7)	0.0284 (5)	0.0239 (6)	0.0066 (5)	0.0079 (5)	−0.0031 (4)
O2	0.0498 (8)	0.0381 (6)	0.0212 (6)	0.0072 (6)	0.0093 (5)	−0.0028 (5)
O3	0.0442 (7)	0.0261 (5)	0.0238 (5)	−0.0026 (5)	0.0131 (5)	−0.0015 (4)
N1	0.0295 (7)	0.0247 (6)	0.0211 (6)	0.0024 (5)	0.0068 (5)	−0.0005 (5)
C1	0.0269 (8)	0.0251 (7)	0.0214 (7)	−0.0029 (6)	0.0103 (6)	−0.0004 (6)
C2	0.0243 (7)	0.0233 (7)	0.0221 (7)	−0.0043 (6)	0.0078 (6)	−0.0001 (5)
C3	0.0297 (8)	0.0274 (7)	0.0222 (7)	−0.0051 (6)	0.0092 (6)	−0.0009 (6)
C4	0.0325 (9)	0.0352 (8)	0.0224 (7)	−0.0026 (7)	0.0049 (6)	0.0048 (6)
C5	0.0284 (8)	0.0325 (8)	0.0341 (9)	0.0014 (6)	0.0067 (7)	0.0075 (7)
C6	0.0320 (9)	0.0275 (7)	0.0335 (8)	0.0018 (6)	0.0122 (7)	−0.0013 (6)
C7	0.0303 (8)	0.0278 (7)	0.0233 (7)	−0.0021 (6)	0.0082 (6)	−0.0016 (6)
C8	0.0299 (8)	0.0271 (7)	0.0240 (7)	0.0047 (6)	0.0089 (6)	0.0026 (6)
C9	0.0293 (8)	0.0315 (8)	0.0231 (7)	0.0030 (6)	0.0029 (6)	0.0005 (6)

### Geometric parameters (Å, °)

**Table:**

O1—C1	1.2573 (17)	C4—C5	1.374 (2)
O2—C3	1.3559 (18)	C4—H4	0.9500
O2—H82	0.90 (2)	C5—C6	1.390 (2)
O3—C9	1.4193 (19)	C5—H5	0.9500
O3—H83	0.90 (2)	C6—C7	1.378 (2)
N1—C1	1.3255 (18)	C6—H6	0.9500
N1—C8	1.4594 (18)	C7—H7	0.9500
N1—H71	0.874 (18)	C8—C9	1.512 (2)
C1—C2	1.484 (2)	C8—H8A	0.9900
C2—C7	1.402 (2)	C8—H8B	0.9900
C2—C3	1.4092 (19)	C9—H9A	0.9900
C3—C4	1.391 (2)	C9—H9B	0.9900
			
C3—O2—H82	106.2 (13)	C6—C5—H5	119.8
C9—O3—H83	109.0 (13)	C7—C6—C5	119.45 (14)
C1—N1—C8	121.13 (12)	C7—C6—H6	120.3
C1—N1—H71	122.0 (12)	C5—C6—H6	120.3
C8—N1—H71	116.8 (12)	C6—C7—C2	121.56 (14)
O1—C1—N1	120.34 (13)	C6—C7—H7	119.2
O1—C1—C2	120.13 (13)	C2—C7—H7	119.2
N1—C1—C2	119.51 (12)	N1—C8—C9	110.64 (12)
C7—C2—C3	117.96 (13)	N1—C8—H8A	109.5
C7—C2—C1	122.61 (13)	C9—C8—H8A	109.5
C3—C2—C1	119.30 (12)	N1—C8—H8B	109.5
O2—C3—C4	117.80 (13)	C9—C8—H8B	109.5
O2—C3—C2	122.16 (13)	H8A—C8—H8B	108.1
C4—C3—C2	120.04 (13)	O3—C9—C8	112.70 (13)
C5—C4—C3	120.54 (14)	O3—C9—H9A	109.1
C5—C4—H4	119.7	C8—C9—H9A	109.1
C3—C4—H4	119.7	O3—C9—H9B	109.1
C4—C5—C6	120.44 (14)	C8—C9—H9B	109.1
C4—C5—H5	119.8	H9A—C9—H9B	107.8
			
C8—N1—C1—O1	2.0 (2)	O2—C3—C4—C5	−179.44 (14)
C8—N1—C1—C2	−176.53 (12)	C2—C3—C4—C5	1.0 (2)
O1—C1—C2—C7	−166.39 (14)	C3—C4—C5—C6	−0.7 (2)
N1—C1—C2—C7	12.2 (2)	C4—C5—C6—C7	0.0 (2)
O1—C1—C2—C3	9.4 (2)	C5—C6—C7—C2	0.3 (2)
N1—C1—C2—C3	−172.01 (13)	C3—C2—C7—C6	0.1 (2)
C7—C2—C3—O2	179.76 (13)	C1—C2—C7—C6	175.95 (13)
C1—C2—C3—O2	3.8 (2)	C1—N1—C8—C9	174.38 (13)
C7—C2—C3—C4	−0.7 (2)	N1—C8—C9—O3	−63.35 (16)
C1—C2—C3—C4	−176.74 (13)		

### Hydrogen-bond geometry (Å, °)

**Table:**

*D*—H···*A*	*D*—H	H···*A*	*D*···*A*	*D*—H···*A*
O2—H82···O1	0.90 (2)	1.73 (2)	2.5570 (15)	150 (2)
O3—H83···O1^i^	0.90 (2)	1.93 (2)	2.8197 (15)	168.3 (19)
N1—H71···O3^ii^	0.874 (18)	2.113 (19)	2.9697 (16)	166.6 (17)
C4—H4···O2^iii^	0.95	2.54	3.4864 (19)	173.
C7—H7···O3^ii^	0.95	2.57	3.4496 (19)	155.

Symmetry codes: (i) *x*, −*y*+1/2, *z*−1/2; (ii) −*x*, −*y*, −*z*; (iii) −*x*+1, −*y*, −*z*+2.
